# Ovarian carcinoma presenting as cutaneous nasal metastasis*

**DOI:** 10.1590/abd1806-4841.20165024

**Published:** 2016

**Authors:** Ana Marta António, João Vitor Alves, João Goulão, Elvira Bártolo

**Affiliations:** 1 Hospital Garcia de Orta – Almada, Portugal

**Keywords:** Neoplasm metastasis, Neoplasms, unknown primary, Ovarian neoplasms

## Abstract

Metastatic ovarian cancer uncommonly presents with skin metastasis. When present, skin metastases of ovarian cancer are usually localized in the vicinity of the primary tumor. We report a case of a 58-year-old woman with a rapid growing erythematous, well-defined nodule localized on the left nasal ala. A skin biopsy was performed and histopathological and immunohistochemical findings were compatible with a cutaneous metastasis of adenocarcinoma. A systematic investigation revealed a bilateral ovarian cystadenocarcinoma associated with visceral dissemination, likely associated with nose cutaneous metastasis. We report a very uncommon case because of the presentation of ovarian carcinoma as cutaneous metastasis. To our knowledge, this atypical localization on the nose has not been described yet in the literature.

## INTRODUCTION

Ovarian carcinoma is the third most common gynecological cancer and it remains the leading cause of death among women who develop cancers of gynecologic origin.^1-3^ Metastatic spread usually involves the peritoneum and the intra-peritoneal structures. The most common site of extra-peritoneal involvement is the lung.^1^ Skin is involved in only 1.9%-5.1% of cases.^2,3^ Metastatic spread to the skin is uncommon and generally occurs in late-stage of disease and its presence invariably carries a poor prognosis.^4^ Usually, its cutaneous localization occurs in the vicinity of the primary tumor, frequently in the abdominal wall.^5^

## CASE REPORT

**Figure 1:**
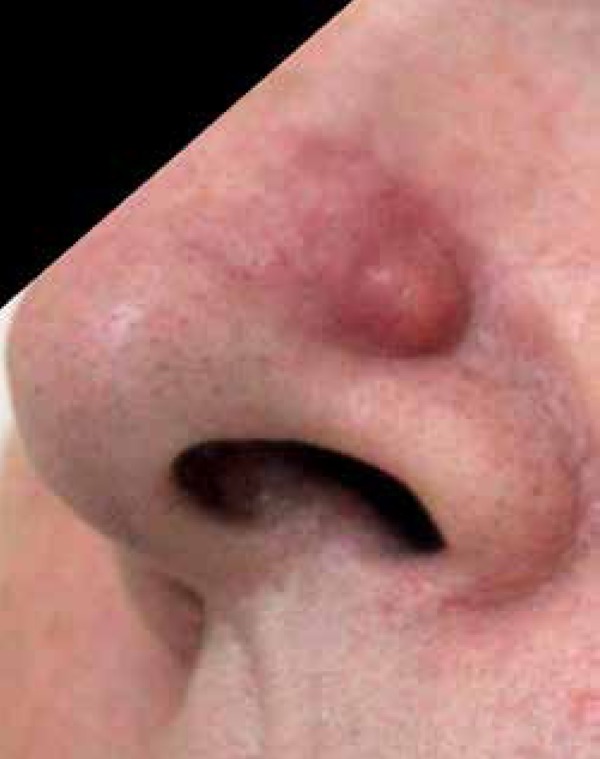
An erythematous and well-defined, 1-cm nodule, covered and surrounded by superficial telangiectasias on the left nasal ala

**Figure 2:**
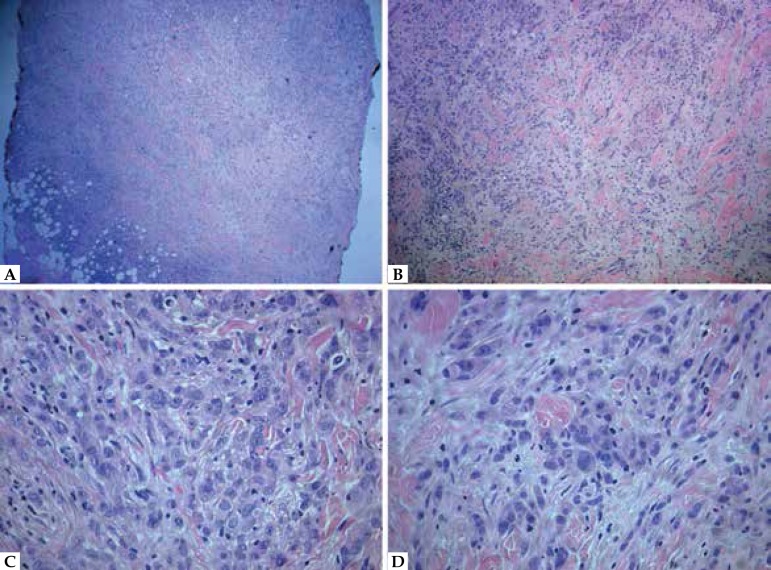
**A)** Dense and infiltrative cellular infiltrate occupying the dermis and hypodermis (hematoxylin and eosin 40x); **B)** Infiltrative cords of epithelioid cells in the dermis (hematoxylin and eosin 100x); **C** and **D)** The infiltrate is composed by pleomorphic epithelioid and some hyperchromatic cells. Mitoses were also seen. (hematoxylin and eosin 400x)

**Figure 3:**
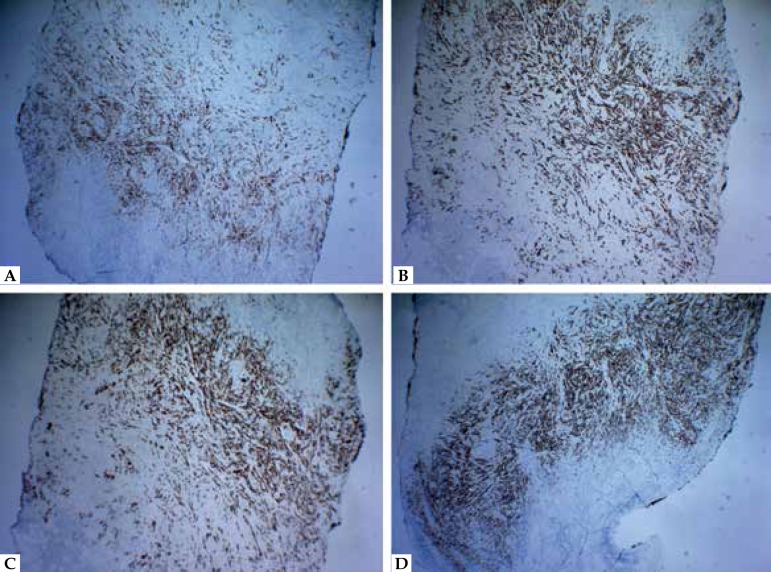
**A)** Positivity staining for CEA (40x); **B)** Positivity of the neoplastic cells for CK AE1/AE3 (40x); **C)** Positivity staining for EMA (40x); **D)** Positivity of the neoplastic cells for CK 7 (40x)

**Figure 4:**
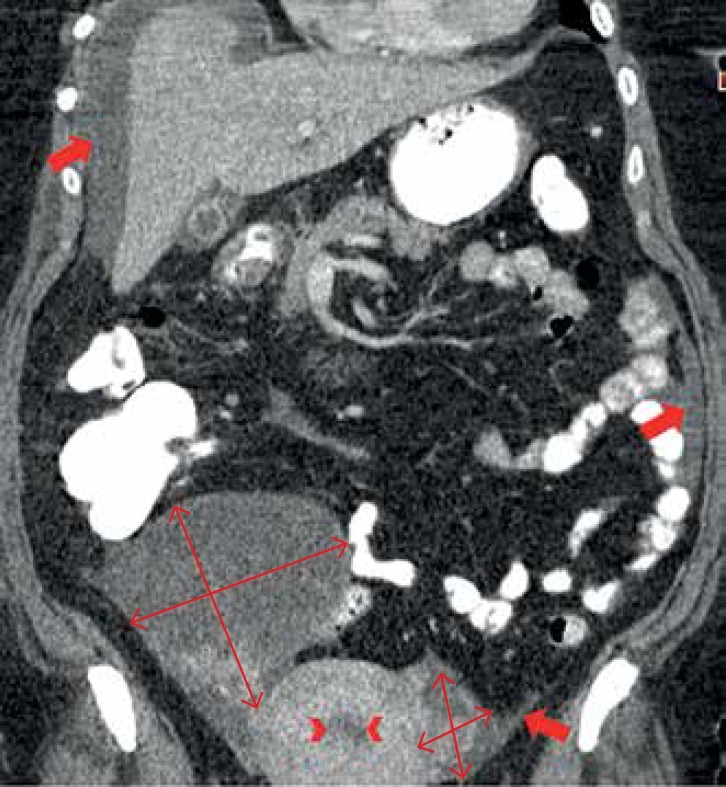
Thoracoabdominopelvic contrast-enhanced CT scan (coronal reconstruction image) demonstrating bilateral expansive lesions in the ovaries (double arrows). They are predominantly cystic, with a solid, hyper-enhancing component, suggesting a bilateral cystadenocarcinoma of the ovary. Endometrial hyperplasia (arrowheads) and ascites (thick arrows) are also seen.

The authors report the case of a 58-year-old woman who was referred to our department due to a 3-month rapid growing and painful nodule located on the nose. Skin examination revealed a solitary, erythematous, well-defined, fibrous-elastic nodule, 1-cm in size, covered and surrounded by superficial telangiectasia, localized on the left nasal ala (Figure 1). Dermoscopic evaluation showed arborizing vessels without any other relevant feature. We observed no lymphadenopathy or palpable hepatosplenomegaly. Differential diagnoses included basal cell carcinoma, cutaneous sarcoidosis, cutaneous b-cell lymphoma and cutaneous metastasis. A skin biopsy was performed and showed dense infiltrative cords of polymorphic epithelioid cells occupying the dermis and hypodermis (Figure 2). Immunohistochemistry revealed positivity staining for cytokeratin (CK) 7, CK AE1/AE3, carcinoembryonic antigen (CEA), and epithelial membrane antigen (EMA) (Figure 3). The immunostaining was negative for CK 20, E-cadherin, gross cystic disease fluid protein (GCDFP)-15, estrogen, and progesterone. These histopathological findings were compatible with a cutaneous metastasis. Despite the undifferentiated histopathological profile, immunohistochemical staining raised the possibilitty of a micro-cystic ductal carcinoma or a metastatic adenocarcinoma. Given these findings, an extensive and systematic clinical and imaging investigation to find the primary carcinoma was performed. The only clinical complaint were an intermittent metrorrhagia associated with a low-grade abdominal pain. Physical exam was unremarkable, including the breast examination and gynecology evaluation. Laboratory analysis revealed a normocytic and normochromic anemia (hemoglobin 11.4 g/L) with no other hematological, biochemical or serological alteration. Our patient was recently submitted to a negative colonoscopy. face por head neck computer tomography (CT) revealed no space occupying the lesion and only showed reactive adenopathies. Mammography and breast ultrasound were normal. Thoracoabdominopelvic computer tomography was also performed and revealed two expansive, predominantly cystic masses in both adnexal areas (with 10x16x11 cm on the right and 4x4x5 cm on the left), with solid hyper-enhancing components, suggesting bilateral ovarian cystadenocarcinoma (Figure 4). There was associated endometrial hyperplasia, malignant ascites and suspicious hepatic nodules. According to the staging system of the International Federation of Gynecology and Obstetrics (FIGO) it was considered a stage IV ovarian cancer.^2,3,6^ The patient was referred to a specialized gynecologic oncology department where she was proposed to initiate palliative chemotherapy. Unfortunately, the patient died three months after the initial diagnosis due to the extent of the malignant disease.

## DISCUSSION

Cutaneous metastases from ovarian cancer are generally nodular lesions, most often located on the abdomen or thorax.^1,3^ Some reports have described metastases in lower extremities, neck and arms.^1,3,4^ To our knowledge, cutaneous nasal metastasis from ovarian cancer has not yet been reported. In this case, the cutaneous metastasis was predominantly undifferentiated. However, given the cutaneous histology and immunohistochemistry, the presence of a metastatic bilateral ovarian carcinoma and the absence of other lesions on the head neck and thoracoabdominopelvic CT, the diagnosis of a cutaneous metastasis from ovarian carcinoma is very likely. Unfortunately, the prognosis of ovarian cancer with cutaneous metastases is poor. Based on the FIGO staging system for ovarian carcinoma, skin metastasis defines stage IV, which also includes distant metastases to pulmonary, liver or splenic parenchyma.^2,6,7^ Generally, the reported median survival after presentation of skin metastases is four months.^3^

In summary, we report a very uncommon case: first, because the presentation of ovarian carcinoma as cutaneous metastasis is rare; second, because, to our knowledge, no other reports have described cutaneous nasal metastasis from ovarian cancer. Thus, when diagnosing unusual and bizarre skin lesions, the possibility of metastatic internal malignancies should be considered, since this could be critical to the management and prognosis of the patient.
